# Medium and feed optimization for fed-batch production of a monoclonal antibody in CHO cells

**DOI:** 10.1186/1753-6561-5-S8-P75

**Published:** 2011-11-22

**Authors:** Nadine Kochanowski, Gaetan Siriez, Sarah Roosens, Laetitia Malphettes

**Affiliations:** 1 Cell Culture Process Development Group, Biological Process Development, UCB Pharma S.A., Braine L’Alleud, 1420, Belgium

## Background

Mammalian cells are used extensively in the production of recombinant proteins, and of monoclonal antibodies (MAbs) in particular. The trend towards avoiding animal-derived components in biopharmaceutical production processes has led to the extensive use of non-animal origin hydrolysates such as plant hydrolysates or yeast hydrolysates. The source of hydrolysates affects cell growth and productivity and may also affect product quality. Accordingly, careful consideration should be given during process and cell culture media development, in order to determine the appropriate type and amount of hydrolysates to be added, for the cell and product at hand.

In this study, we assessed the impact of several hydrolysate additives and chemically defined (CD) commercial feeds on MAb titers, MAb average specific productivity (average Qp), cell viabilities and metabolite profiles in suspension cultures of recombinant CHO cells expressing a monoclonal antibody in shake flasks and 2 L bioreactors.

## Materials and methods

Initial experiments were performed using chemically defined culture medium in 125 mL shake flasks with 50 mL working volume. CHO cells were seeded at 0.3x10^6^ viable cells/mL and incubated at 140 rpm, 36.5°C and 5% CO_2_. 2L stirred tank bioreactors (Sartorius) were carried out for 14 days in a fed-batch mode in a chemically defined medium supplemented with chemically defined feeds and hydrolysates. Glucose was maintained between 1 and 6 g/L. At the day of harvest the clarification was performed by depth filtration. Analysis of daily samples included determinations of cell viability, cell density, metabolites, osmolality and product titer. Product concentration of the supernatant samples was quantified using Octet QK and Protein A high performance liquid chromatography (HPLC). Protein characterization of Protein-A purified samples were profiled by reduced and non reduced SDS PAGE. Isoelectric focusing (IEF) analysis of Protein-A purified MAb was carried out using a iCE280 IEF Analyzer. Aggregates and monomers proportion were determined by using size exclusion chromatography. Acidic and basic species were characterized using anion exchange (AEX) HPLC. Oligosaccharides were cleaved enzymatically using N-Glycanase, then labeled with 2-aminobenzamide and analyzed by HPLC using an amide column and a fluorescent detector.

## Results

Several chemically defined feeds and hydrolysates were assessed on CHO cells expressing a monoclonal antibody in fed-batch mode. The performance of the developed process was compared to an existing in-house platform process.

**Figure 1:**
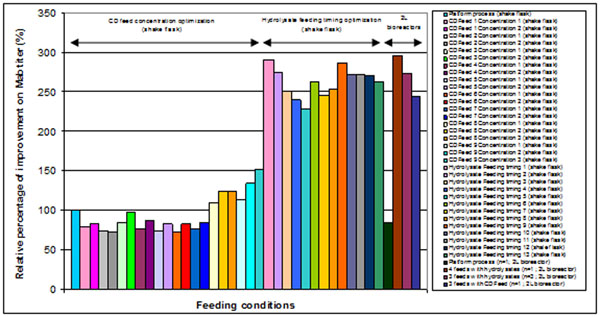
Relative percentage of improvement on MAb titer. Note: Platform process in shake flask was used as the 100% reference for all the calculations of relative percentage of improvement on Mab titer measured the day of harvest

Nine different chemically defined feeds were assessed and added at different concentrations (Table 1). Among the feeds tested, addition of CD Feed 8 and 9 brought a 150 % improvement on MAb titer on the day of harvest compared to the platform process. All the MAb titers measured were ranging from 2 g/L to 6 g/L (Figure 1).

**Table 1:** Chemically defined feeds and hydrolysates tested

Supplier	Commercial feed name	Feed name in the poster
ThermoFischer	Cell Boost 1	CD Feed 1
ThermoFisher	Cell Boost 2	CD Feed 2
ThermoFisher	Cell Boost 3	CD feed 3
ThermoFischer	Cell Boost 4	CD Feed 4
ThermoFischer	Cell Boost 5	CD Feed 5
ThermoFischer	Cell Boost 6	CD Feed 6
Life Tech	CHO Feed A	CD Feed 7
Life Tech	CHO Feed B	CD Feed 8
Life Tech	CHO Feed C	CD Feed 9
BD Biosciences	Yeast Extract	Hydrolysate 1
BD Biosciences	Yeastolate	Hydrolysate 2
BD Biosciences	Select Phytone	Hydrolysate 3
BD Biosciences	Ultrapep Soy	Hydrolysate 4
Sheffield	HyPep 1510	Hydrolysate 5
Sheffield	HyPep 4605	Hydrolysate 6
BD Biosciences	3 g/L Yeast Extract + 3.25 g/L Yeastolate	Hydrolysate combination 1
BD Biosciences	3.5 g/L Yeast Extract + 2.75 g/L Yeastolate	Hydrolysate combination 2
BD Biosciences	4 g/L Yeast Extract + 2.25 g/L Yeastolate	Hydrolysate combination 3
BD Biosciences	4.5 g/L Yeast Extract + 1.75 g/L Yeastolate	Hydrolysate combination 4
BD Biosciences	5 g/L Yeast Extract + 1.25 g/L Yeastolate	Hydrolysate combination 5
BD Biosciences	3 g/L Yeast Extract + 5.1 g/L Yeastolate	Hydrolysate combination 6
BD Biosciences	3.5 g/L Yeast Extract + 4.6 g/L Yeastolate	Hydrolysate combination 7
BD Biosciences	4 g/L Yeast Extract + 4.1 g/L Yeastolate	Hydrolysate combination 8
BD Biosciences	4.5 g/L Yeast Extract + 3.6 g/L Yeastolate	Hydrolysate combination 9
BD Biosciences	5 g/L Yeast Extract + 3.1 g/L Yeastolate	Hydrolysate combination 10

Six different hydrolysates were assessed at different concentrations in fed-batch mode (Table 1). Among the feeds tested, addition of hydrolysate 1 and hydrolysate 2 showed an improvement of 175% and 167% respectively on MAb yield.

To identify potential synergies between hydrolysates, the best hydrolysates from previous experiments were selected and were tested in combination at different ratios on CHO cell cultures (Table 1). Antibody concentration at harvest was 290% higher with some of the hydrolysate combinations.

Based on feed combination optimization results, the number of bolus feeds and the feed addition timing were then fine-tuned using the best hydrolysate combination. Reducing the number of bolus feeds enabled to reduce ammonia and osmolality while maintaining a high MAb titer (Figure 1). Moreover, under these conditions, cell viabilities were maintained above 80% throughout the culture (data not shown).

Based on experimental results obtained in shake flasks, the best hydrolysate combination (3 feeds and 4 feeds) and CD feed were assessed on CHO cells cultured in 2 L stirred tank bioreactors. Cell growth and cell metabolism were monitored daily throughout the cultures in bioreactors. By feeding the cultures with hydrolysates, addition of 3 or 4 bolus feeds enabled to attain similar maximum viable cell count. Addition of chemically defined feed led to a 30% higher maximum viable cell count. Cell viabilities were maintained at acceptable values throughout the cultures in the established culture conditions. Lactate profiles were similar independently of the feeding regime. Decreasing the number of hydrolysate feeds enabled to maintain osmolality and ammonia at acceptable concentrations for CHO cell growth and product quality. Cell growth performance and metabolism profiles observed in 2 L bioreactors were comparable to those observed in shake flasks.

Product titers have been measured throughout the fed-batch cultures with the Octet QK system. The MAb titers were in a 2-6 g/L range for all the tested feeding regimes at the day of harvest. A combination of hydrolysates and a chemically defined feed supplementation showed an improvement of 296% and 245% on MAb concentration at the day of harvest in comparison to the platform process (Figure 1). MAb average specific productivity (Qp) was increased by 700% and 360% by adding 4 feeds of hydrolysates and chemically defined feed respectively. Decreasing the number of hydrolysate feeds showed a slight decrease on MAb titer and on Qp.

Product quality attributes were determined on cell culture clarified fluids after Protein-A purification. Reduced and non reduced SDS electrophoresis, isoelectric focusing (IEF) analysis, gel permeation HPLC, size exclusion (SEC) chromatography, anion exchange HPLC (AEX) have been used to characterize the Protein-A purified MAb. Product quality data was comparable for all the feeding regimes tested in 2 L bioreactors.

## Conclusions

Hydrolysate combination additions significantly improved MAb production in comparison to single hydrolysate addition or chemically defined feeds. Number of bolus feeds and feeding timing optimization enabled us to improve the process robustness taking into account the impact of feeding strategy on cell metabolism and product quality. The feeding regimes established in shake flasks led to similar culture performance in 2 L bioreactors.

